# Different Prognostic Implications of ^18^F-FDG PET Between Histological Subtypes in Patients With Cervical Cancer: Erratum

**DOI:** 10.1097/01.md.0000484045.74107.88

**Published:** 2016-05-20

In the article “Different Prognostic Implications of ^18^F-FDG PET Between Histological Subtypes in Patients With Cervical Cancer”,^1^ which appeared in Volume 95, Issue 9 of *Medicine*, funding information was originally omitted. The article has since been corrected online.
